# Serum bilirubin and chronic obstructive pulmonary disease (COPD): a systematic review

**DOI:** 10.1186/s12890-021-01395-9

**Published:** 2021-01-20

**Authors:** David M. MacDonald, Ken M. Kunisaki, Timothy J. Wilt, Arianne K. Baldomero

**Affiliations:** 1 Division of Pulmonary, Allergy, Critical Care, and Sleep Medicine, Department of Medicine, University of Minnesota, Suite 350, VCRC, 401 East River Road, Minneapolis, MN 55455 USA; 2 Section of Pulmonary, Allergy, Critical Care, and Sleep Medicine, Department of Medicine, Minneapolis Veterans Affairs Health Care System, Minneapolis, MN USA; 3 Center for Care Delivery and Outcomes Research and the Section of General Medicine, Minneapolis Veterans Affairs Health Care System, Minneapolis, MN USA; 4 Department of Medicine, University of Minnesota, Minneapolis, MN USA

**Keywords:** Pulmonary disease, chronic obstructive, Bilirubin, Antioxidants, Systematic review

## Abstract

**Background:**

Bilirubin is a potent antioxidant and higher serum bilirubin levels have been associated with improved COPD outcomes. We performed a systematic review to evaluate the association between serum bilirubin levels and lung function (FEV_1_), prevalence/incidence of COPD, acute exacerbations of COPD, respiratory health status, and mortality.

**Methods:**

MEDLINE® and Embase were searched using Ovid® (search updated October 1st, 2019). We included studies that measured serum bilirubin levels and outcomes of interest in adults with or without underlying lung disease. We excluded studies of those with liver disease or drug-induced elevations in bilirubin. We used the Newcastle–Ottawa scale to assess individual study risk of bias (ROB) and the US Agency for Healthcare Research and Quality—Evidence Based Practice tool to assess overall strength of evidence (SOE). Two authors independently determined eligibility, performed data abstraction, assessed ROB, and determined SOE.

**Results:**

Thirteen studies (5 low risk of bias, 3 moderate and 5 high risk) were included. We found low strength of evidence for the association between higher bilirubin levels and lower risk of acute exacerbations of COPD (2 studies), mortality (3 studies), COPD diagnosis (4 studies), and lung function (FEV_1_) (8 studies). We found insufficient evidence on the relationship between serum bilirubin and respiratory health status/exercise capacity (1 study) and airflow obstruction (FEV_1_/FVC ratio) (4 studies).

**Conclusion:**

Higher bilirubin levels may be associated with lower mortality and improved COPD outcomes. Randomized trials are needed to evaluate the effect of medications that raise serum bilirubin on COPD outcomes.

*PROSPERO registration*: CRD42019145747.

## Background

Chronic obstructive pulmonary disease (COPD) is among the leading causes of death worldwide [1]. Although the mortality rate for COPD has been decreasing in some countries, it has continued to rise in others [2]. In addition to mortality, COPD is a leading cause of respiratory-associated disability, health care utilization, and economic burden, with much of the cost and morbidity driven by acute exacerbations [3, 4].

Bilirubin, the main metabolic end-product of heme degradation, is a potent anti-oxidant that scavenges peroxyl radicals and inhibits membrane-bound nicotinamide adenine dinucleotide phosphate oxidase (NADPH) oxidase, which is a large intracellular source of reactive oxygen species [5–9]. Oxidative stress is increased in stable COPD, with further increases during acute exacerbations of COPD (AECOPD) [10]. NADPH oxidase plays a role in lung signaling and deleterious remodeling, and has been implicated in a variety of lung diseases, including COPD [11, 12]. Observational studies have previously suggested that higher serum bilirubin concentrations, but still within the normal range of serum bilirubin (what we have termed ‘benign elevations’), are associated with decreased COPD diagnosis, lower risk of AECOPD, slower rate of lung function decline, and lower all-cause mortality [13–19]. However, other studies have found no effect of serum bilirubin on similar outcomes [20–22]. The pathogenesis of COPD is complex, but oxidative stress is commonly observed in AECOPD and has been associated with faster decline in the forced expiratory volume in 1 s (FEV_1_) [12]. Therefore, the potent anti-oxidant effect of bilirubin could be a novel therapeutic intervention [9].

We evaluated the evidence on potential protective benefits of benign elevations in serum bilirubin in COPD. Our key questions (KQ) were:

- *KQ1* Is serum bilirubin associated with better lung function as measured by FEV_1_?
- *KQ2* Is serum bilirubin associated with lower prevalence/incidence of COPD and/or better (higher) FEV_1_/FVC ratio?
- *KQ3* Is serum bilirubin level associated with improved clinically relevant outcomes (respiratory events/exacerbations, respiratory symptoms, respiratory health status, and mortality)?

## Methods

This systematic review was conducted according to Preferred Reporting Items for Systematic Reviews and Meta-analyses (PRISMA) standards [23]. Our protocol was registered in PROSPERO (registration number CRD42019145747).

### Data sources and searches

We searched MEDLINE® and Embase databases using Ovid® (search updated October 1st, 2019) for peer-reviewed, English language studies. The search strategy for the MEDLINE® search is provided in Additional file 1: Appendix 1.

### Study selection

Two authors (AKB and DMM) independently reviewed each study at the abstract and full-text level to determine eligibility. Disagreements were resolved through discussion and evaluation by a third reviewer, if needed.

Reports were eligible if they were randomized or observational (cohort, cross-sectional, case control) studies that enrolled participants greater than 18 years of age. We did not exclude studies based on pulmonary function or pulmonary diagnoses and included studies conducted in both general populations and in samples restricted to those with obstructive lung disease. We excluded case reports and case series, studies published in only abstract form, and those without a full text publication in English. We did not have a numerical cutoff for bilirubin levels because there are no defined criteria for what constitutes a pathologic elevation in bilirubin, but we excluded studies evaluating elevations in serum bilirubin in the context of hepatobiliary diseases and drug-induced liver injury. Eligible studies reported serum bilirubin levels and any outcome of interest related to our three key questions.

### Outcome measures

We examined clinically important outcomes by cross sectional measures and longitudinal measures. Clinical effect sizes are included in Additional file 1: Appendix 2.

### Data extraction and quality assessment

Data extraction was independently completed by two reviewers (DMM and AKB). We extracted data on study characteristics (first author, year published, sample size, and study population), inclusion criteria, serum bilirubin levels, outcome measurements, methodology of outcome assessments, and covariates included in modeling the outcome of interest. If a study included both cross-sectional and longitudinal analyses, the longitudinal analysis results were preferentially selected for this systematic review.

We assessed risk of bias for observational studies using the Newcastle–Ottawa Scale (NOS), a validated tool used to assess quality of nonrandomized studies [24, 25]. The NOS uses a star system to assess a study on three main domains: selection of study groups (four star scale), comparability of study groups (two star scale), and ascertainment of either exposure or outcome of interest (three star scale). There are no accepted criteria for assigning risk of bias based upon the number of stars. We a priori defined studies as “low” risk of bias if they met all nine stars across the assessment criteria. “Moderate” risk of bias was assigned to studies that met seven or eight stars. “High” risk of bias was assigned to studies that met six or fewer stars, if the reviewers could not ascertain star criteria for a study, and for studies with n ≤ 200. The Newcastle–Ottawa Scale is shown in Additional file 1: Appendix 3.

### Data analysis

Data synthesis for this review was limited to qualitative summary due to high clinical heterogeneity (variation in study population and outcomes measured) and methodological heterogeneity (variation in study design, exposure measures, outcome measures, and model covariates). We were unable to perform quantitative analysis of FEV_1_ (mL/year) because the 95% confidence interval (CI) was only available for one study.

### Overall strength of evidence

Two reviewers independently assessed the strength of evidence based on methods guidance provided by the Evidence Based Practice Center (EPC) established by the U.S. Agency for Healthcare Research and Quality (AHRQ), adapted for exposure (serum bilirubin level) rather than an intervention [26]. A complete description of the assessment of strength of evidence is included in Additional file 1: Appendix 2.

## Results

**Fig. 1:**
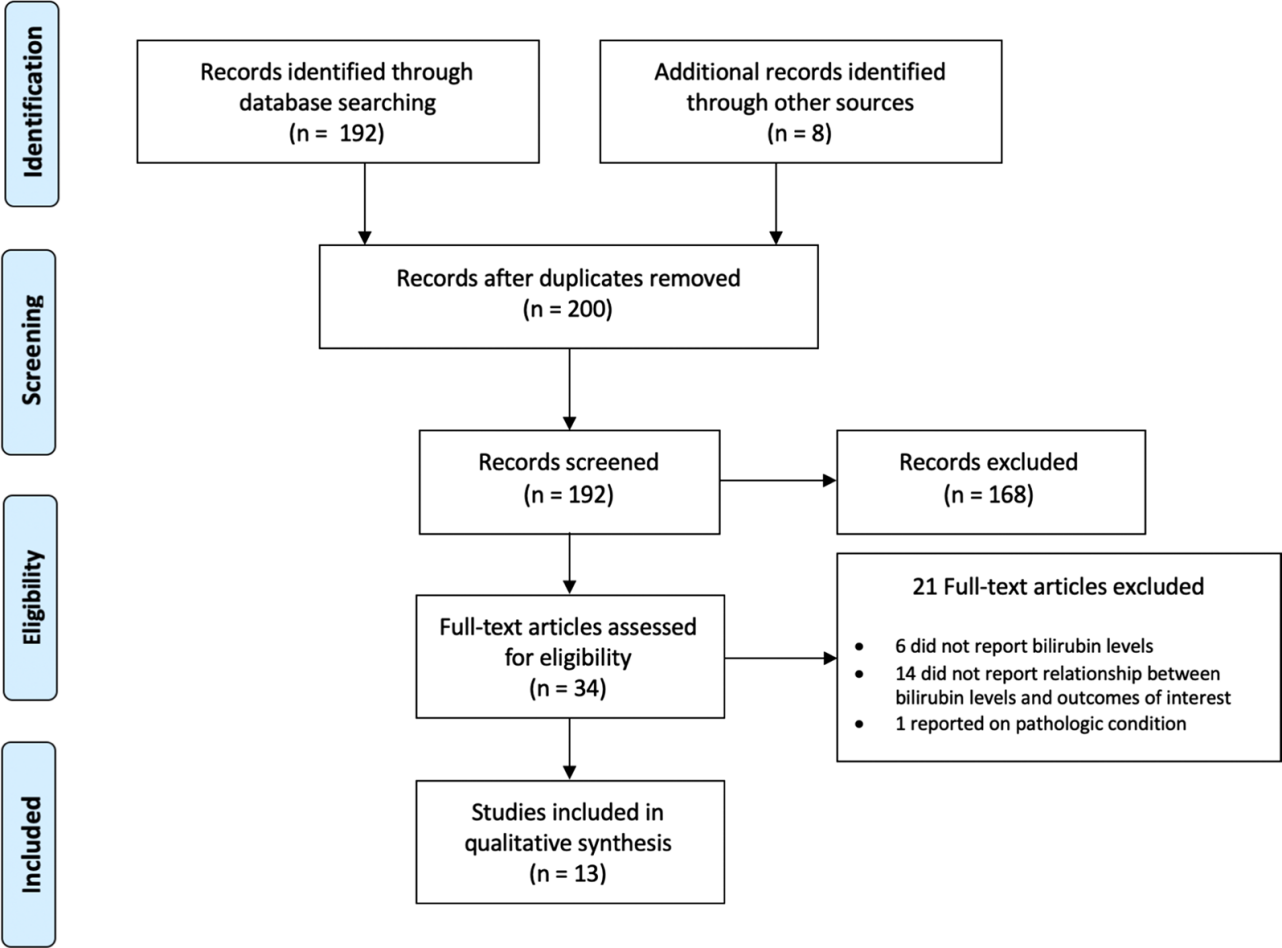
PRISMA flow diagram

We identified 192 articles for title and abstract screening and 8 articles through additional sources (Fig. 1). Thirteen articles met inclusion criteria [13–22, 27–29], all observational studies. Two studies were secondary analyses of randomized trials but were analyzed as observational studies [13, 20]. Study characteristics including author and year, country, number of participants, description of the population (sex, current smoking status, race), FEV_1_, baseline serum bilirubin levels, upper limit of bilirubin levels, outcomes of interest, and study level risk of bias are listed in Additional file 1: Table S1. Studies excluded after full-text review are listed in Additional file 1: Appendix 4.

Four [13, 16, 19, 21] of the 13 studies [13–22, 27–29] were from North America (United States and Canada), four from Europe [14, 17, 18, 29], four from East Asia (China or South Korea) [15, 22, 27, 28], and one was from a cohort of participants in 20 countries [20] (Additional file 1: Table S1). Five studies were rated low risk of bias [13, 15, 17, 19, 20], three moderate risk of bias [14, 18, 27], and five high risk of bias [16, 21, 22, 28, 29] (Additional file 1: Table S2). Sample size ranged from 131 to 504,206 [17, 22]. The mean age was 54.4 years (10 studies reporting) [13–19, 21, 27, 28] and 58.8% were men (12 studies reporting) [13–22, 27, 29]. All studies reported on current smoking status, and the weighted proportion of current smokers was 22.8%; two studies excluded smokers [21, 22]. The mean bilirubin level was 0.62 mg/dL (range 0.37–0.76 mg/dL) (8 studies reporting) [13–17, 19, 21, 27]. Three studies [13, 17, 27] excluded participants with a bilirubin level above 1.75 mg/dL for women and 2.34 mg/dL for men, and one study [18] excluded participants with a bilirubin level > 0.99 mg/dL. Five studies [13, 16, 19–21] reported race; one study (38.2% Black, 17.5% Latino/Hispanic, 9.6% Asian, 33.7% White, and 1.0% other) had broad representation [20], one was 40.4% non-Hispanic White [16], and the remainder were predominantly White (range 77–100%) [13, 19, 21].

Seven studies evaluated the association between serum bilirubin and clinical outcomes of interest [13, 17, 19, 22, 27–29]. Eight studies [13–16, 18, 20, 21, 27] reported the association between serum bilirubin and FEV_1_ and four studies [15, 18, 20, 27] evaluated the association between serum bilirubin and FEV_1_/FVC ratio.

**Fig. 2:**
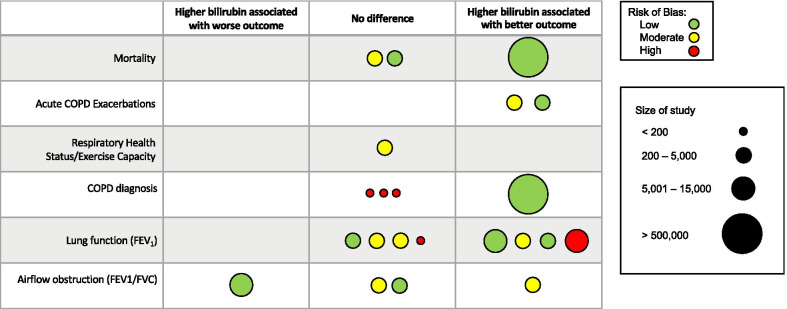
Summary of measured outcomes. Each row represents an outcome of interest. Columns are divided by statistical significance, with the no difference column representing studies that found no statistically significant relationship for the outcome of interest. Individual studies are represented by circles and are colored green for low risk of bias, yellow for moderate risk of bias, and red for high risk of bias. The size of each circle is representative of the number of participants in the study, but not directly proportional. Each study can be represented by more than one circle if it investigated more than one outcome of interest

Figure 2 illustrates the summary of the measured outcomes, stratified by statistical significance. Additional file 1: Table S3 provides the overall assessment of strength of evidence for all outcomes evaluated.

### Clinical outcomes

Seven studies evaluated clinically important outcomes and are shown in Table 1 [13, 17, 19, 22, 27–29].

**Table 1:** Associations between serum bilirubin and clinically relevant outcomes

First author, year	Measure	Outcome	Model covariates
Apperly 2015 [13]	Logistic regression of relationship between bilirubin quintile and mortality	OR of mortality in quintile 1 vs 5 (reference) 1.15 (95% CI 0.80–1.65; p = 0.32) OR of respiratory mortality in quintile 1 vs 5 (reference) 1.59 (95% CI 0.50–5.11; p = 0.58)	Age, sex, BMI, race, and pack-years smoked
Brown 2017 [19]	Time dependent multivariable Cox proportional hazards analysis for time to first AECOPD	STATCOPE (development and calibration): aHR per log(10) increase in bilirubin = 0.89 (95% CI 0.74–1.09; p = 0.26) MACRO (validation): aHR per log(10) increase in bilirubin = 0.80 (95% CI 0.67–0.94; p = 0.008)	Treatment assignment, sex, race, BMI, chronic bronchitis, supplemental O2 use, SGRQ score, inhaler use, steroid or antibiotic use in last year Treatment assignment, sex, race, BMI, bronchitis, supplemental O2 use, SGRQ score, inhaler use, steroid or antibiotic use in last year
Horsfall 2011 [17]	Poisson regression between overall mortality and bilirubin levels Poisson regression between diagnosis of COPD and bilirubin levels	Men: aIRR per 0.1 mg/dL increase in bilirubin 0.97 (95% CI 0.97–0.98; p < 0.001) Women: aIRR per 0.1 mg/dL increase in bilirubin 0.97 (95% CI 0.96–0.98; p < 0.001) Men: aIRR per 0.1 mg/dL increase in bilirubin 0.94 (95% CI 0.93–0.95; p < 0.001) Women: aIRR per 0.1 mg/dL increase in bilirubin 0.94 (95% CI 0.92–0.95; p < 0.001)	Age, BMI, systolic blood pressure, smoking status, alcohol intake, social deprivation score Treatment assignment, sex, race, BMI, bronchitis, supplemental O2 use, SGRQ score, inhaler use, steroid or antibiotic use in last year
Lee 2018 [22]	Modified Poisson regression for risk of COPD in high vs low bilirubin	aRR 1.17 (95% CI 0.80–1.71; p = 0.431)	Age, sex, BMI
Leem 2019 [27]	Cox regression model for mortality Linear mixed model and generalized estimating equations for number of exacerbations per year, 6 MW, CAT, and SGRQ	HR = 1.60 (95% CI 0.65–3.97; p = 0.311) # Exacerbations/year: β = 0.62 (SE 0.18; p = 0.001) 6 MW: β = 20.5 (SE 12.2; p = 0.094) CAT: β = 0.4 (SE 1.2; p = 0.746) SGRQ: β = 2.9 (SE 2.5; p = 0.261)	age, sex, BMI, Smoking, baseline FEV_1_
Milevoj Kopcinovic 2016 [29]	Bilirubin in COPD vs controls by Mann–Whitney or t-test	6.4 (IQR 5.2–8.3) vs 7.1 (IQR 5.5 to 10.9); p = 0.102	No difference so not included in additional analysis
Wei 2015 [28]	Bilirubin in COPD vs controls by one-way ANOVA	No significant difference, values not reported	None

*OR* odds ratio, *BMI* body mass index, *aIRR* adjusted incidence rate ratio, *HR* hazard ratio, *FEV*_*1*_ forced expiratory volume in 1 s, *6MW* 6 min walk, *CAT* COPD assessment test, *SGRQ* St. George’s Respiratory Questionnaire, *AECOPD* acute exacerbation of COPD, *aHR* adjusted hazard ratio, *CI* confidence interval, *SE* standard error, *STATCOPE* Simvastatin for the Prevention of Exacerbations in Moderate-to-Severe COPD, *MACRO* Macrolide Azithromycin to Prevent Rapid Worsening of Symptoms Associated with Chronic Obstructive Pulmonary Disease

#### Mortality

Three studies evaluated mortality [13, 17, 27]. One large (n = 504,206) primary care based medical record analysis with low risk of bias found a statistically significant adjusted incidence rate ratio (aIRR) for both men (0.97, 95% CI 0.97–0.98) per 0.1 mg/dL increase in serum bilirubin] and women (0.97, 95% CI 0.96–0.98) per 0.1 mg/dL increase in serum bilirubin] [17]. The other two studies, both in cohorts of persons with obstructive lung disease, found no association between serum bilirubin and all-cause mortality [13, 27]. One of these studies, in a North American longitudinal cohort of smokers with obstructive spirometry, found no association with all-cause mortality [odds ratio (OR) in quintile 1 vs 5 (reference) 1.15, 95% CI 0.80–1.65] or respiratory disease-related mortality [OR in quintile 1 vs 5 (reference) 1.31, 95% CI 0.42–4.09], but there was an association with coronary heart disease-related mortality (OR 2.20 in quintile 1 vs. quintile 5, 95% CI 0.95 to 5.14; p for trend = 0.03) [13].

We found an inverse association of bilirubin levels with all-cause mortality (“low” strength of evidence). Though the largest study showed a statistically significant association between higher serum bilirubin and lower mortality [17], the overall body of evidence lacked precision and consistency.

#### Acute exacerbations of COPD

Two studies evaluated the association between serum bilirubin and AECOPD [19, 27]. Both found higher serum bilirubin was associated with significantly lower rates of AECOPD [19, 27]. One low ROB study was a secondary analysis of two randomized trials of participants with COPD enriched for participants prone to exacerbations. The model was developed in one cohort (n = 853), where bilirubin was not a significant predictor of COPD exacerbation (adjusted hazard ratio per log_10_ increase in bilirubin = 0.89; 95% CI 0.74–1.09, p = 0.26) but in the validation cohort (n = 1018) the relationship between higher serum bilirubin and time to first AECOPD was significant (adjusted hazard ratio per log_10_ increase in bilirubin = 0.80; 95% CI 0.67–0.94, p = 0.008) [19]. The other study (n = 535), which had moderate risk of bias, was in a cohort of patients with obstructive lung disease and found that higher serum bilirubin was associated with lower rates of AECOPD [estimated mean from regression 0.62 (standard error 0.18; p = 0.001)] [27].

We found an inverse association between bilirubin levels and AECOPD (“low” strength of evidence). Though both studies found a statistically significant association between higher serum bilirubin and lower rates of AECOPD, one study found a statistically significant relationship only in the validation cohort, not the development cohort and the other had a moderate risk of bias [19, 27]. There were no data specific to the association between serum bilirubin and risk of hospitalization for AECOPD.

#### Respiratory health status and exercise capacity

One moderate ROB study evaluated respiratory health status (CAT and SGRQ) and exercise capacity (6-min walk test) and found no significant association between serum bilirubin and any of these measures [27]. We determined that there was insufficient evidence on the association of bilirubin with respiratory health status and exercise capacity.

#### COPD diagnosis

Four studies evaluated the association between serum bilirubin and COPD diagnosis [15, 17, 20, 27]. One large (n = 504,206) longitudinal cohort study with low risk of bias found a small but statistically significant decrease in the aIRR of COPD diagnosis based on EMR coding with increasing levels of bilirubin [for men aIRR 0.94 (0.93, 0.95) per 0.1 mg/dL increase in bilirubin; for women aIRR 0.94 (0.92, 0.95) per 0.1 mg/dL increase in bilirubin] [17]. Three studies were small, cross-sectional, and had high risk of bias; none found a significant relationship [22, 28, 29]. We found an inverse association between bilirubin levels and COPD diagnosis. We assigned “low strength evidence” based upon the moderate overall bias, inconsistent effects, and imprecision of reported outcomes.

#### Lung function (FEV_1_)

Results for studies evaluating the association between serum bilirubin and FEV_1_ are shown in Table 2. Among the three studies (all low ROB) [13, 15, 20] that evaluated longitudinal change in FEV_1_, two found a statistically significant relationship. One was a general population cohort with a mean follow-up of 5.4 years (FEV_1_ declined 13.09 mL/yr slower for every 1 unit increase in natural log of bilirubin) and the other was a longitudinal cohort of smokers with a mean FEV_1_/FVC ratio of < 0.7 with up to 9 years of follow-up (FEV_1_ declined 57.0 mL/yr in the highest quintile of bilirubin and 66.3 mL/year in the lowest) [13, 15]. The study that did not find a statistically significant relationship was from a smaller cohort of young, HIV positive adults who were followed for a median of 3.9 years [20].

**Table 2:** Associations between serum bilirubin and FEV_1_

First author, year	Measure	Outcome	Model covariates
*Longitudinal studies*			
Apperley 2015 [13]	Linear regression of relationship between bilirubin quintile and FEV_1_ decline years 2–5	FEV_1_ decline 57.0 (SD 70.7) mL/year in quintile 5 vs 66.3 (SD 64.8) mL/year in quintile 1 (p for trend = 0.001)	Age, sex, BMI, race, FEV_1_ at baseline, LMCR, and pack-years smoked
Leem 2018 [15]	Linear regression between ln(bilirubin) and FEV_1_ decline mL/yr	β = − 13.09 (p < 0.001)	Age, sex, BMI, FEV_1_ at baseline, smoking status
MacDonald 2019 [20]	Linear regression between log(2) bilirubin and FEV_1_ mL/yr	β = -2.1 (95% CI − 8.6 to 4.4; p = 0.53)	Age, sex, race, region, smoking status, treatment group, CD4 T-cell count, and HIV-RNA
*Cross-sectional studies*			
Horsfall 2014 [14]	Mixed linear regression of relationship between ln (bilirubin) and FEV_1_, 2 stage least squares	β = 133 (95% CI 37–228; p = 0.007)	Age, sex, height, smoking status, region of birth
Schunemann 1997 [21]	Linear regression of FEV_1_% predicted against bilirubin	β = 10.35 (SE 5.90; p = 0.082)	Age, height, gender, smoking status (former vs lifelong non-smoker)
Yang 2015 [16]	Pearson’s correlation for relationship between total bilirubin and FEV_1_	r/t = 0.203 (p < 0.001)	None—included in MV analysis but details of model not included
Leem 2019 [27]	Linear mixed model for relationship between serum bilirubin and FEV_1_	Estimated mean = 0.04 (SE 0.08; p = 0.607)	Age, sex, BMI, smoking
Curjuric 2014 [18]	Linear regression of relationship between ln(bilirubin) and FEV_1_	β = 13.8 (95% CI − 15.5 to 43.2; p = 0.356)	Sex, age, height, weight, education, study area, ever smoking, total pack years

*FEV*_*1*_ forced expiratory volume in 1 s, *BMI* body mass index, *LMCR* logarithm of methacholine reactivity, *MV* multivariate, *ln* natural log, *HIV* human immunodeficiency virus, *CI* confidence interval, *SE* standard error

Five studies [14, 16, 18, 21, 27], 3 moderate ROB [14, 18, 27] and 2 high ROB [16, 21] evaluated cross-sectional associations between bilirubin and FEV_1._ Two studies found a statistically significant relationship between higher FEV_1_ and higher serum bilirubin [14, 16]. One low risk of bias cohort sample from England, Wales, and Scotland [14] evaluated 5,362 people born in the same week in 1946 and the other high risk of bias study [21] was from a nationally representative cohort in the United States. Three found no significant relationship between FEV_1_ and serum bilirubin levels [18, 21, 27].

We found an association between higher bilirubin levels and improved lung function (FEV_1_). We assigned a “low” strength of evidence due to the moderate overall risk of bias of the eight studies evaluated, clinically small effect, inconsistent effects, and imprecision of the outcome (unreported or wide confidence intervals).

#### Airflow obstruction (FEV_1_/FVC ratio)

Four studies evaluated the association between serum bilirubin and FEV_1_/FVC ratio, a spirometric marker of obstructive lung disease [15, 18, 20, 27] (Table 3). Three of the studies were longitudinal [15, 20, 27]. One longitudinal study with low risk of bias found an association between higher baseline bilirubin levels and faster decline in FEV_1_/FVC ratio [ß for % decline in FEV_1_/FVC per 1 unit change in natural log of bilirubin (mg/dL) = 1.69; p < 0.001] [15]; two found no significant relationship [20, 27]. A cross-sectional population-based analysis with moderate risk of bias found a significant association between higher bilirubin levels and less airflow obstruction [ß for % decline in FEV_1_/FVC per 1 unit increase in natural log in bilirubin (μmol/L) 0.5; 95% CI 0.1–1.0, p = 0.012] [18]. There was insufficient evidence to evaluate the relationship between airflow obstruction and serum bilirubin.

**Table 3:** Associations between serum bilirubin and airflow obstruction (FEV_1_/FVC ratio)

First author, year	Measure	Outcome	Model covariates
Curjuric 2014 [18]	Linear regression of relationship between ln(bilirubin) and FEV_1_/FVC ratio	β = 0.5 (95% CI 0.1–1.0; p = 0.012)	Sex, age, height, weight, education, study area, ever smoking, total pack years
MacDonald 2019 [20]	Linear regression of log(2) bilirubin on FEV_1_/FVC slope	β = − 0.7 (− 95% CI 1.6–0.2; p = 0.12)	Age, sex, race, region, smoking status, treatment group, CD4 T-cell count, and HIV-RNA
Leem 2018 [15]	Linear regression between ln(bilirubin) and decline in FEV_1_/FVC ratio (annual)	β = 1.69 (p < 0.001)	Age, sex, BMI, FEV_1_/FVC at baseline, smoking status
Leem 2019 [27]	Linear mixed model for relationship between serum bilirubin and FEV_1_/FVC	Estimated mean = − 0.45 (SE 1.72; p = 0.792)	Age, sex, BMI, smoking

*COPD* chronic obstructive pulmonary disease, *aIRR* adjusted incidence rate ratio, *BMI* body mass index, *FEV*_*1*_ forced expiratory volume in 1 s, *FVC* forced vital capacity, *HIV* human immunodeficiency virus, *aRR* adjusted rate ratio, *ANOVA* analysis of variance, *CI* confidence interval, *SE* standard error

## Discussion

This report provides the first systematic review of the relationship between “benign” elevations in serum bilirubin levels and COPD clinical outcomes and lung function. Our analysis of data from 13 observational studies found an association between higher serum bilirubin and reduced mortality and improved COPD outcomes and lung function measures (low strength evidence). The effect based on a priori thresholds was deemed small in absolute magnitude. Due to clinical and methodologic heterogeneity and data reporting limitations we were unable to perform a meta-analysis. Instead, we presented a summary of findings across studies and outcomes. The strength of evidence for associations varied by outcome from “insufficient” for quality of life, exercise capacity, and FEV1/FVC ratio to “low” for the relationship between higher serum bilirubin and risk of AECOPD, lung function (FEV_1_), COPD diagnosis, and mortality.

Though we found low strength of evidence, our most consistent finding was the association between serum bilirubin and AECOPD. This is particularly intriguing given the increased oxidative stress observed during and after AECOPD, and the potent anti-oxidant effects of bilirubin [5–8, 12]. Only one of these studies evaluated the correlation between bilirubin and other anti-oxidant markers. They found a statistically significant, but negligible correlation (Pearson’s correlation coefficient 0.19, p < 0.05) between bilirubin and the Trolox equivalent antioxidant capacity (TEAC) assay, which has been used as a summary measurement of antioxidant activity [21]. *N*-acetylcysteine and carbocisteine, which have anti-oxidant and anti-inflammatory properties, may have a role in reduction of AECOPD [30, 31]. Though antioxidants have been disappointing when used for the prevention of lung function decline, increased dietary intake of antioxidants may be associated with better lung function outcomes [32, 33]. Whether bilirubin levels can be manipulated to provide a similar benefit has not been investigated.

We identified gaps in the existing literature that limited our conclusions. The study designs and populations were heterogenous, preventing quantitative analyses. For example, of the three longitudinal studies analyzing the relationship between serum bilirubin and FEV_1_, one was a cohort of smokers with mild to moderate airflow obstruction from the Lung Health Study, one was a Korean cohort which excluded those with preexisting lung disease, and one was a cohort from a randomized controlled study of young, HIV positive participants [13, 15, 20]. These studies also used different methods to quantify bilirubin (e.g. quintile, natural log or log_2_ transformation). Lastly, one of these studies did not report a 95% confidence interval for the regression coefficient [15]. Similar methodologic and population differences were present for the other outcomes of interest. COPD diagnosis and mortality findings were also influenced mostly by one large study [17]. Though we assigned clinical effect sizes to the outcomes of interest, we were unable to utilize these to assign an overall clinical effect size due to heterogeneity in reporting of outcomes and analysis methods. Lastly, this analysis may be limited by reporting bias, where negative analyses within these studies were not reported, and by the post hoc nature of these studies.

The studies included in this review were all observational, and therefore, we were unable to establish causality. No randomized controlled trials have assessed the effectiveness and harms of increasing bilirubin levels on adults with or at risk for COPD-related morbidity and mortality. Bilirubin excretion is mediated by UDP-glucuronosyl transferase-1A1 (UGT1A1), the enzyme that conjugates bilirubin for excretion into the bile. Polymorphisms in UGT1A1 account for approximately 10–40% of the variation in serum bilirubin levels and these polymorphisms may be associated with improved respiratory function [34–36]. Gilbert’s syndrome, a genetic cause of benign bilirubin elevations, is caused by a deficiency in UGT1A1. Inhibition of UGT1A1 is a possible target to raise bilirubin levels, and there are already existing medications, such as the HIV antiretroviral atazanavir, that inhibit UGT1A1 and cause reversible elevations in bilirubin [37].

Though not the focus of this systematic review, Horsfall and colleagues examined the relationship between genetic changes underlying Gilbert’s syndrome and respiratory outcomes. They found associations with higher FEV_1_ and FVC, a relationship which was strongest among heavy smokers who would be at greatest risk of developing COPD [14]. In an analysis of UK Biobank data, Horsfall et al. also found that genetic changes associated with higher bilirubin levels are associated with a decreased risk of lung cancer, a relationship that was also strongest in heavy smokers [36]. The degree of increase in bilirubin and duration of therapy that would be needed to improve COPD and respiratory health outcomes remain unknown, and would need to be investigated in interventional trials. We excluded studies of pathologic elevations in bilirubin as our goal was to analyze whether benign elevations in bilirubin were a possible therapeutic target. Though several of the included studies eliminated participants with bilirubin levels greater than 1.75 mg/dL for women and 2.34 mg/dL for men [13, 17, 27] or greater than 0.99 mg/dL for all participants [18], there is no broadly accepted upper limit of normal for bilirubin levels.

This review highlighted areas for further study. The relationship between serum bilirubin levels and patient-centered outcomes such as exercise capacity and respiratory health status should be evaluated in future studies; this has only been done in one study thus far [27]. Randomized controlled trials of interventions that increase serum bilirubin levels should be considered, especially in those prone to AECOPD because they are at greater risk of future events and are in need of additional novel therapies to reduce their AECOPD risk.

## Conclusion

Higher serum bilirubin within the normal range may be associated with lower mortality and risk of COPD exacerbations, a reduced incidence in COPD diagnosis and improved lung function Interventional trials are needed to test if medications that raise serum bilirubin levels can safely improve COPD outcomes.

## Supplementary information

**Additional file 1:** Appendices and supplemental tables.

## Data Availability

All data generated or analyzed during this study are included in this published article [and its supplementary information files].

## Abbreviations

AECOPD: Acute exacerbation of chronic obstructive pulmonary disease
AHRQ: U.S. Agency for Healthcare Research and Quality
aIRR: Adjusted incidence rate ratio
CAT: COPD assessment test
CI: Confidence interval
COPD: Chronic obstructive pulmonary disease
EMR: Electronic medical record
EPC: Evidence based practice center
FEV_1_: Forced expiratory volume in 1 s
FVC: Forced vital capacity
HIV: Human immunodeficiency virus
KQ: Key question
NADPH: Nicotinamide adenine dinucleotide phosphate
NOS: Newcastle–Ottawa Scale
OR: Odds ratio
PRISMA: Preferred reporting items for systematic reviews and meta-analyses
ROB: Risk of bias
SGRQ: St. George’s Respiratory Questionnaire
SOE: Strength of evidence

## References

[1] GBD Causes of Death Collaborators (2018). Global, regional, and national age-sex-specific mortality for 282 causes of death in 195 countries and territories, 1980–2017: a systematic analysis for the Global Burden of Disease Study 2017. Lancet.

[2] Mathers CD, Loncar D (2006). Projections of global mortality and burden of disease from 2002 to 2030. PLoS Med.

[3] Vos T, Flaxman AD, Naghavi M, Lozano R, Michaud C, Ezzati M, Shibuya K, Salomon JA, Abdalla S, Aboyans V, Abraham J, Ackerman I, Aggarwal R, Ahn SY, Ali MK, Almazroa MA, Alvarado M, Anderson HR, Anderson LM, Andrews KG, Atkinson C, Baddour LM, Bahalim AN, Barker-Collo S, Barrero LH, Bartels DH, Basáñez MG, Baxter A, Bell ML, Benjamin EJ (2012). Years lived with disability (YLDs) for 1160 sequelae of 289 diseases and injuries 1990–2010: a systematic analysis for the Global Burden of Disease Study 2010. Lancet.

[4] Ehteshami-Afshar S, Fitzgerald JM, Doyle-Waters MM, Sadatsafavi M (2016). The global economic burden of asthma and chronic obstructive pulmonary disease. Int J Tuberc Lung Dis.

[5] Neuzil J, Stocker R (1994). Free and albumin-bound bilirubin are efficient co-antioxidants for alpha-tocopherol, inhibiting plasma and low density lipoprotein lipid peroxidation. J Biol Chem.

[6] Sedlak TW, Saleh M, Higginson DS, Paul BD, Juluri KR, Snyder SH (2009). Bilirubin and glutathione have complementary antioxidant and cytoprotective roles. PNAS.

[7] Stocker R, Glazert AN, Ames BN (1987). Antioxidant activity of albumin-bound bilirubin. Proc Natl Acad Sci.

[8] Brandes RP, Weissmann N, Schröder K (2010). NADPH oxidases in cardiovascular disease. Free Radic Biol Med.

[9] Stocker R, Yamamoto Y, McDonagh AF, Glazer AN, Ames BN (1987). Bilirubin is an antioxidant of possible physiological importance. Science.

[10] John A, Mcguinness A, Sapey E (2017). Oxidative stress in COPD: sources, markers, and potential mechanisms. J Clin Med.

[11] Harijith A, Natarajan V, Fu P (2017). The role of nicotinamide adenine dinucleotide phosphate oxidases in lung architecture remodeling. Antioxidants.

[12] Kirkham PA, Barnes PJ (2013). Oxidative stress in COPD. Chest.

[13] Apperley S, Park HY, Holmes DT, Man SFP, Tashkin D, Wise RA, Connett JE, Sin DD (2015). Serum bilirubin and disease progression in mild COPD. Chest.

[14] Horsfall LJ, Hardy R, Wong A, Kuh D, Swallow DM (2014). Genetic variation underlying common hereditary hyperbilirubinaemia (Gilbert’s syndrome) and respiratory health in the 1946 British birth cohort. J Hepatol.

[15] Leem AY, Kim HY, Kim YS, Park MS, Chang J, Jung JY (2018). Association of serum bilirubin level with lung function decline: a Korean community-based cohort study. Respir Res.

[16] Yang HF, Kao TW, Wang CC, Peng TC, Chang YW, Chen WL (2015). Serum white blood cell count and pulmonary function test are negatively associated. Acta Clin Belgica Int J Clin Lab Med.

[17] Horsfall LJ, Rait G, Walters K, Swallow DM, Pereira SP, Nazareth I, Petersen I (2011). Serum bilirubin and risk of respiratory disease and death. JAMA.

[18] Curjuric I, Imboden M, Adam M, Bettschart RW, Gerbase MW, Künzli N, Rochat T, Rohrer L, Rothe TB, Schwartz J, Stolz D, Tschopp JM, Von Eckardstein A, Kronenberg F, Probst-Hensch NM (2014). Serum bilirubin is associated with lung function in a Swiss general population sample. Eur Respir J.

[19] Brown KE, Sin DD, Voelker H, Connett JE, Niewoehner DE, Kunisaki KM (2017). Serum bilirubin and the risk of chronic obstructive pulmonary disease exacerbations. Respir Res.

[20] Macdonald DM, Zanotto AD, Collins G, Baker JV, Czarnecki M, Loiza E, Nixon DE, Papastamopoulos V, Wendt CH, Wood R, Kunisaki KM (2019). Associations between baseline biomarkers and lung function in HIV-positive individuals. AIDS.

[21] Schunemann HJ, Muti P, Freudenheim JL, Armstrong D, Browne R, Klocke RA, Trevisan M (1997). Oxidative stress and lung function. Am J Epidemiol.

[22] Lee H, Hong Y, Lim MN, Bak SH, Kim MJ, Kim K, Kim WJ, Park HY (2018). Inflammatory biomarkers and radiologic measurements in never-smokers with COPD: a cross-sectional study from the CODA cohort. Chron Respir Dis.

[23] Moher D, Shamseer L, Clarke M, Ghersi D, Liberati A, Petticrew M, Shekelle P, Stewart L, PRISMA-P Group (2015). Preferred reporting items for systematic review and meta-analysis protocols (PRISMA-P) 2015 statement. Syst Rev.

[24] Wells G, Shea B, O’Connell D, Peterson J, Welch V, Losos M, P T. Newcastle-Ottawa Quality Assessment Scale. http://www.ohri.ca/programs/clinical_epidemiology/oxford.asp.

[25] Stang A (2010). Critical evaluation of the Newcastle-Ottawa scale for the assessment of the quality of nonrandomized studies in meta-analyses. Eur J Epidemiol.

[26] Berkman ND, Lohr KN, Ansari MT, Balk EM, Kane R, McDonagh M, Morton SC, Viswanathan M, Bass EB, Butler M, Gartlehner G, Hartling L, McPheeters M, Morgan LC, Reston J, Sista P, Whitlock E, Chang S (2015). Grading the strength of a body of evidence when assessing health care interventions: an EPC update. J Clin Epidemiol.

[27] Leem AY, Kim YS, Lee J, Kim T, Kim HY, Oh YM, Do LS, Jung JY (2019). Serum bilirubin level is associated with exercise capacity and quality of life in chronic obstructive pulmonary disease. Respir Res.

[28] Wei J, Zhao H, Fan G, Li J (2015). Bilirubin treatment suppresses pulmonary inflammation in a rat model of smoke-induced emphysema. Biochem Biophys Res Commun.

[29] Milevoj Kopčinović L, Domijan AM, Posavac K, Čepelak I, Žanić Grubišić T, Rumora L (2016). Systemic redox imbalance in stable chronic obstructive pulmonary disease. Biomarkers.

[30] Cazzola M, Calzetta L, Page C, Jardim J, Chuchalin AG, Rogliani P, Matera MG (2015). Influence of N-acetylcysteine on chronic bronchitis or COPD exacerbations: a meta-analysis. Eur Respir Rev.

[31] Zheng JP, Kang J, Huang SG, Chen P, Yao WZ, Yang L, Bai CX, Wang CZ, Wang C, Chen BY, Shi Y, Liu CT, Chen P, Li Q, Wang ZS, Huang YJ, Luo ZY, Chen FP, Yuan JZ, Yuan BT, Qian HP, Zhi RC, Zhong NS (2008). Effect of carbocisteine on acute exacerbation of chronic obstructive pulmonary disease (PEACE Study): a randomised placebo-controlled study. Lancet.

[32] Garcia-Larsen V, Potts JF, Omenaas E, Heinrich J, Svanes C, Garcia-Aymerich J, Burney PG, Jarvis DL (2017). Dietary antioxidants and 10-year lung function decline in adults from the ECRHS survey. Eur Respir J.

[33] Decramer M, Rutten-Van Mölken M, Dekhuijzen PNR, Troosters T, Van Herwaarden C, Pellegrino R, Van Schayck CPO, Olivieri D, Del Donno M, De Backer W, Lankhorst I, Ardia A (2005). Effects of N-acetylcysteine on outcomes in chronic obstructive pulmonary disease (Bronchitis Randomized on NAC Cost-Utility Study, BRONCUS): a randomised placebo-controlled trial. Lancet.

[34] Chen G, Ramos E, Adeyemo A, Shriner D, Zhou J, Doumatey AP, Huang H, Erdos MR, Gerry NP, Herbert A, Bentley AR, Xu H, Charles BA, Christman MF, Rotimi CN (2012). UGT1A1 is a major locus influencing bilirubin levels in African Americans. Eur J Hum Genet.

[35] Lin J, Cupples LA, Wilson PWF, Heard-costa N, Donnell CJO (2003). Evidence for a gene influencing serum bilirubin on chromosome 2q telomere: a genomewide scan in the Framingham Study. Am J Hum Genet.

[36] Horsfall LJ, Burgess S, Hall I, Nazareth I, Horsfall LJ, Burgess S, Hall I (2020). Lung cancer Genetically raised serum bilirubin levels and lung cancer : a cohort study and Mendelian randomisation using UK Biobank. Thorax.

[37] Roy-Chowdhury J, Roy-Chowdhury N, Listowsky I, Wolkoff AW (2017). Drug and drug abuse associated hyperbilirubinemia: experience with atazanavir. Clin Pharmacol Drug Dev.

