# Supplementary material for: Serum bilirubin and chronic obstructive pulmonary disease (COPD): a systematic review
Source: BMC Pulm Med. 2021 Jan 20;21:33. doi: 10.1186/s12890-021-01395-9 (PMC7816373; doi:10.1186/s12890-021-01395-9)
Supplement: Supplementary file 1 — Additional file 1: Appendices and supplemental tables. [file 12890_2021_1395_MOESM1_ESM.docx]

**Additional files**

**Serum Bilirubin and Chronic Obstructive Pulmonary Disease (COPD):**

**A Systematic Review**

David M. MacDonald^1,2^, Ken M. Kunisaki^1,2^, Timothy J. Wilt^3,4^, Arianne K. Baldomero^1,2^

**Authors’ affiliations:**

^1^Division of Pulmonary, Allergy, Critical Care, and Sleep Medicine, Department of Medicine, University of Minnesota, Minneapolis, MN, USA

^2^Division of Pulmonary, Allergy, Critical Care, and Sleep Medicine, Department of Medicine, Minneapolis Veterans Affairs Health Care System, Minneapolis, MN, USA

^3^Center for Care Delivery and Outcomes Research and the Section of General Medicine, Minneapolis Veterans Affairs Health Care System, Minneapolis, MN, USA

^4^Department of Medicine, University of Minnesota, Minneapolis, MN, USA

**Additional files:**

Additional file 1: Appendix 1. Search strategy

Additional file 1: Appendix 2.. Supplementary methods

Additional file 1: Appendix 3. Newcastle-Ottawa Scale

Additional file 1: Appendix 4. Studies excluded in full text review

Additional file 1: Table 1. Study characteristics and risk of bias

Additional file 1: Table 2. Risk of bias assessment

Additional file 1: Table 3. Strength of evidence

Additional file 1: References

**Additional file 1: Appendix 1. Search Terms (includes both Medline® and Embase®)**


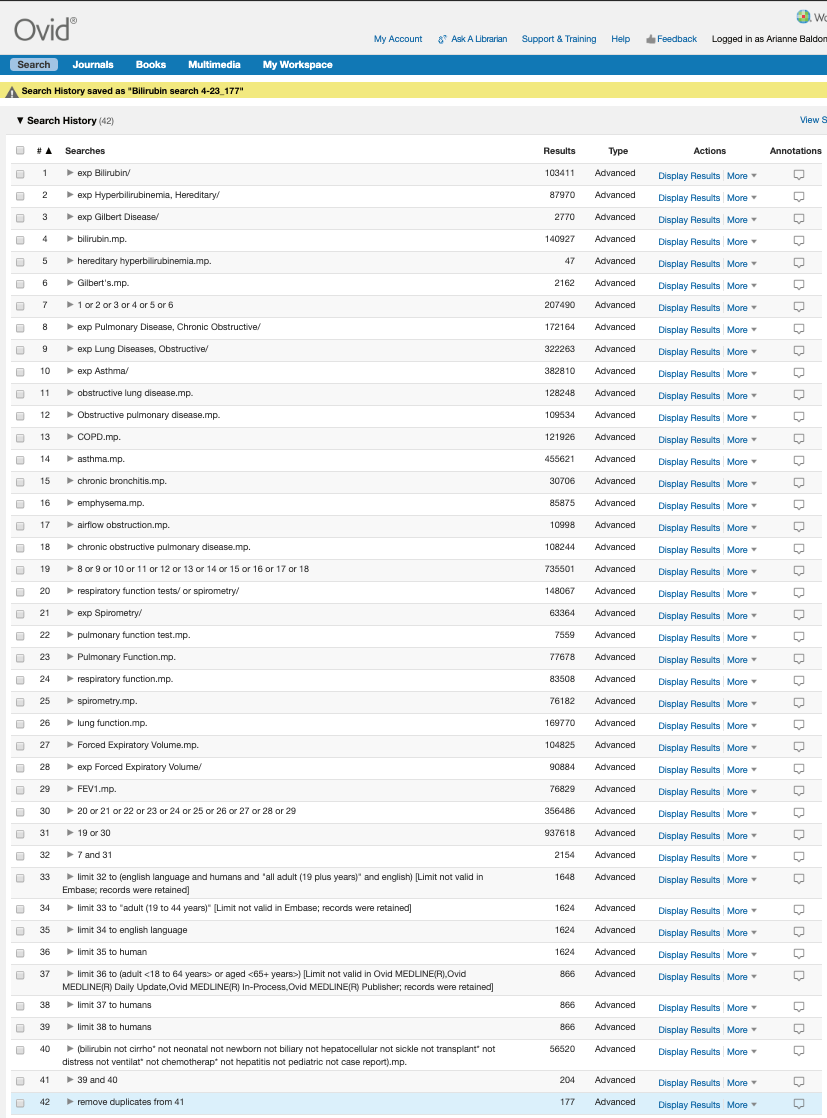


**Additional file 1: Appendix 2. Supplementary methods**

**Outcomes Measures.**

The minimal clinically important difference for the St. George’s Respiratory Questionnaire (SGRQ) is ≥4 points, and for the COPD Assessment Test (CAT) is ≥2 points, and we defined a clinically important difference as above these thresholds [1, 2]. The minimal clinically important improvement in the 6-minute walk distance in COPD has been defined as both 42 and 54 meters, and we defined a clinically important improvement as greater than 48 meters [3]. Recent trials targeting reductions in AECOPD have been powered to detect a relative reduction in exacerbation risk of 15% to 20% [4–6]. We defined little or no clinical effect size as <5%, a small clinical effect size as a change of 5 to <10%, a moderate clinical effect size of 10-20%, and a large clinical effect size of >20%. For the diagnosis of COPD, we defined little or no clinical effect as < 0.05 relative risk difference, a small clinical effect size as a 0.05 to 0.10, a moderate clinical effect size as >0.10 to 0.15, and a large clinical effect size as > 0.15.Finally, for mortality, we defined a small clinical effect size as <1%, a moderate clinical effect size as 1-2%, and a large clinical effect size as >2%.

We examined lung function as measured by cross sectional measures or longitudinal decline in FEV_1_. Large clinical trials have been powered to find a difference of as low as 7.5 mL/year, and normal lung function decline is generally thought to be between 20 and 40 mL/yr, with faster rates of decline with increasing age[7, 8]. Therefore, we defined little or no effect to be a change of <20 mL/year, a small clinical effect size to be 20-30 mL/year, a moderate size to be >30-40 mL/year, and a large effect size >40 mL/year.

We examined obstruction on spirometry (FEV_1_/FVC ratio) by cross-sectional and longitudinal measures. There are no established minimal clinically important differences for either measure. In a large study of general population cohorts the standard deviation of the FEV_1_/FVC ratio was 0.09 [9]. We defined little or no clinical effect as a change in the FEV_1_/FVC ratio of <0.03, a small clinical effect size to be 0.03 to 0.06, a moderate clinical effect size to be >0.06 to 0.09, and a large clinical effect size to be >0.09.

**Strength of Evidence**

The strength of evidence was assessed as high, medium, low, or insufficient based on the following domains: study limitations (risk of bias; low, moderate, or high), consistency (consistent, inconsistent, or unknown), directness (direct or indirect), and precision (precise or imprecise). The strength of evidence was graded as “high” if the body of evidence had few or no deficiencies (findings are stable); “moderate” if the body of evidence had some deficiencies (findings are likely to be stable, but some doubt remains); “low” if the body of evidence had major and/or numerous deficiencies (additional evidence is needed); and “insufficient” if there was no evidence available or the body of evidence had unacceptable deficiencies, precluding a conclusion.

**Additional file 1: Appendix 3. Newcastle-Ottawa Scale**


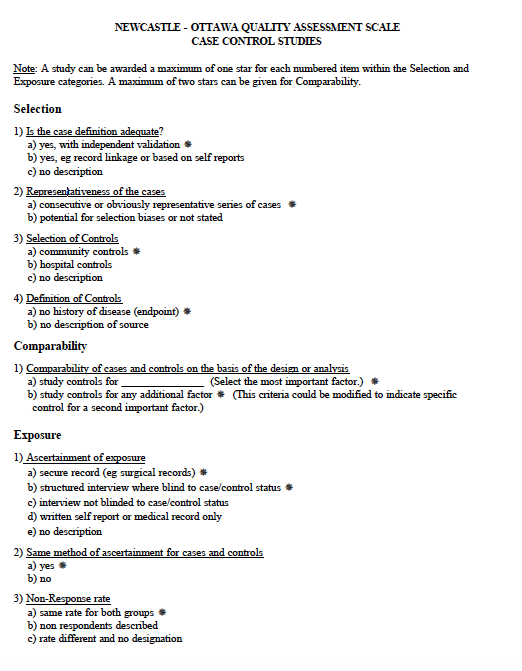


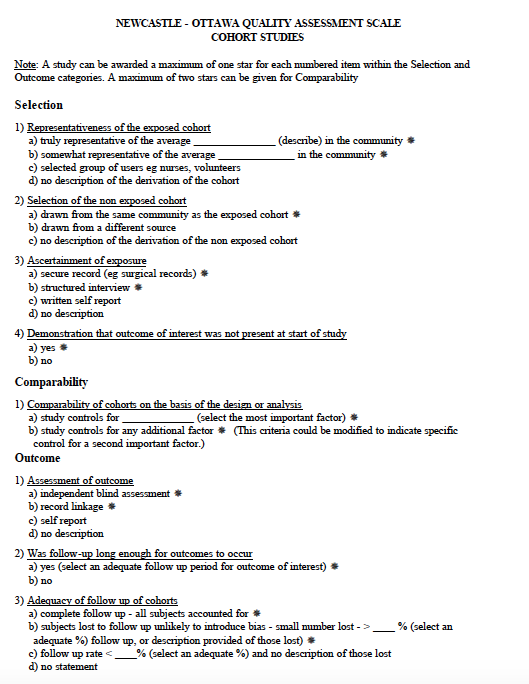


**Additional file 1: Appendix 4. Studies Excluded in Full-Text Review**

1. Lipowicz A, Szklarska A, Malina RM. Allostatic load and socioeconomic status in Polish adult men. J Biosoc Sci. 2014;46(2):155-167.

2. Frost-Pineda K, Liang Q, Liu J, et al. Biomarkers of potential harm among adult smokers and nonsmokers in the total exposure study. Nicotine Tob Res. 2011;13(3):182-193.

3. Brott DA, Goodman MJ, Hermann RP, et al. Are laboratory parameter (biomarker) values similar to the healthy volunteer reference range in all patient populations? Drug Des Devel Ther. 2018;12:2757-2773.

4. Katz DA, Isaacson JD, Han L, Drajesk JF, Comer GM, Heath-Chiozzi ME. Clinical observation of liver chemistry abnormalities in asthmatics. Curr Drug Saf. 2009;4(3):173-180.

5. von Schonfeld J, Breuer N, Zotz R, et al. Liver function in patients with pulmonary emphysema due to severe alpha-1-antitrypsin deficiency (Pi ZZ). Digestion. 1996;57(3):165-169.

6. Oh JY, University K, Khan A, et al. Markers and metabolites related to inflammation in bronchiectasis with airflow limitation vs. Chronic obstructive pulmonary disease. Biomedical Research (India). 2019;29(14):2925-2931.

7. Stepniewski M, Kolarzyk E, Zagrodzki P, et al. Pattern recognition methods in evaluation of the structure of the laboratory data biominerals, antioxidant enzymes, selected biochemical parameters, and pulmonary function of welders. Biol Trace Elem Res. 2003;93(1-3):39-46.

8. Wu X, Wen CP, Ye Y, et al. Personalized Risk Assessment in Never, Light, and Heavy Smokers in a prospective cohort in Taiwan. Scientific Reports. 2016;6:36482.

9. Misso NL, Brooks-Wildhaber J, Ray S, Vally H, Thompson PJ. Plasma concentrations of dietary and nondietary antioxidants are low in severe asthma. Eur Respir J. 2005;26(2):257-264.

10. Lee SJ, Kim HJ, Kim JY, et al. Serum Albumin and Disease Severity of Non-Cystic Fibrosis Bronchiectasis. Respir Care. 2017;62(8):1075-1084.

11. Breimer LH, Wannamethee G, Ebrahim S, Shaper AG. Serum bilirubin and risk of ischemic heart disease in middle-aged British men. Clin Chem. 1995;41(10):1504-1508.

12. Mellstrom D, Rundgren A, Jagenburg R, Steen B, Svanborg A. Tobacco smoking, ageing and health among the elderly: a longitudinal population study of 70-year-old men and an age cohort comparison. Age Ageing. 1982;11(1):45-58.

13. Song JU, Hwang J, Ahn JK. Serum uric acid is positively associated with pulmonary function in Korean health screening examinees. Mod Rheumatol 2017;27:1057–1065.

14. Bae CY, Kang YG, Piao MH, Cho B, Cho KH, Park YK, Yu BY, Lee SW, Kim MJ, Lee SH, Kim YJ, Kim DH, Kim JS, Oh JE. Models for estimating the biological age of five organs using clinical biomarkers that are commonly measured in clinical practice settings. Maturitas 2013;75:253–260.

15. Chen WL, Wang CC, Wu LW, Kao TW, Chan JYH, Chen YJ, Yang YH, Chang YW, Peng TC. Relationship between lung function and metabolic syndrome. PLoS One 2014;9:1–7.

16. Horváth I, Donnelly LE, Kiss A, Paredi P, Kharitonov SA, Barnes PJ. Raised levels of exhaled carbon monoxide are associated with an increased expression of heme oxygenase-1 in airway macrophages in asthma: A new marker of oxidative stress. Thorax 1998;53:668–672.

17. Morris CR, Kim HY, Klings ES, Wood J, Porter JB, Trachtenberg F, Sweeters N, Olivieri NF, Kwiatkowski JL, Virzi L, Hassell K, Taher A, Neufeld EJ, Thompson AA, Larkin S, Suh JH, Vichinsky EP, Kuypers FA, Neufeld E. Dysregulated arginine metabolism and cardiopulmonary dysfunction in patients with thalassaemia. Br J Haematol 2015;169:887–898.

18. Mouronte-Roibás C, Leiro-Fernández V, Ruano-Raviña A, Ramos-Hernández C, Casado-Rey P, Botana-Rial M, García-Rodríguez E, Fernández-Villar A. Predictive value of a series of inflammatory markers in COPD for lung cancer diagnosis: a case-control study. Respir Res 2019;20:1–10.

19. Nakamura E, Miyao K. Further Evaluation of the Basic Nature of the Human Biological Aging Process Based on a Factor Analysis of Age-related Physiological Variables. Journals Gerontol Ser A Biol Sci Med Sci 2003;58:B196–B204.

20. Russo P, Tomino C, Santoro A, Prinzi G, Proietti S, Kisialiou A, Cardaci V, Fini M, Magnani M, Collacchi F, Provinciali M, Giacconi R, Bonassi S, Malavolta M. FKBP5 rs4713916: A potential genetic predictor of interindividual different response to inhaled corticosteroids in patients with chronic obstructive pulmonary disease in a real-life setting. Int J Mol Sci 2019;20:.

21. Singh B, Ghosh N, Saha D, Sarkar S, Bhattacharyya P, Chaudhury K. Effect of doxycyline in chronic obstructive pulmonary disease - An exploratory study. Pulm Pharmacol Ther 2019;58:101831.

**Additional file 1: Table 1. Study characteristics and risk of bias for included studies**

| Author/Year  Country | N | Population | Baseline characteristics  Male n (%)  Current smokers n(%)  Age  Race  FEV1  Serum bilirubin  Upper limit of bilirubin for inclusion | Follow-up duration | Outcome(s) measured | Risk of Bias |
| --- | --- | --- | --- | --- | --- | --- |
| **Longitudinal studies** | | | | | | |
| Apperley 2015  United States and Canada | 4680 | Lung Health Study: longitudinal cohort of smokers with FEV_1_ percent predicted 55-90 and FEV_1_/FVC < 0.70 | Male: 2956 (63.2)  Current smokers: 2530 (54.1)  Age (mean): 53.5 years^1^  Race: white - 4510 (96.4)  FEV1 (mean): 2.73 L^1^  Serum Bilirubin – mean (SD): 0.43 (0.19) mg/dL  UL Bilirubin: women: 1.75 mg/dL, men: 2.34 mg/dL | Up to 9 years^2^ | FEV_1_ mL/yr  Mortality | Low |
| Brown 2017  United States and Canada | STATCOPE = 853  MACRO =  1018 | Secondary analysis of RCTs; STATCOPE (development) and MACRO (validation) trials. Inclusion criteria for both trials: age ≥ 40 years, FEV_1_/FVC <0.7, FEV_1_ <80% predicted. Both studies were enriched for those at risk of AECOPD. | ﻿STATCOPE:  Male: 479 (56)  Current smokers: 257 (30)  Age – mean (SD): 62 (8)  Race: Black 177(21), White 651(77), Other 20(2)  FEV1 % pred – mean (SD): 42 (18)  Serum Bilirubin – mean (SD): 0.65 (0.30) mg/dL  UL Bilirubin: no UL  MACRO:  Male – n(%): 606 (60)  Current smokers – n(%): 222 (22)  Age – mean (SD): 66 (9)  Race: Black 138(14), White 830(82), Other 50(5)  FEV1 % pred – mean (SD): 40 (16)  Serum Bilirubin – mean (SD): 0.64 (0.29) mg/dL  UL Bilirubin: no UL | ﻿STATCOPE: median (IRQ): 635 (329 – 990) days  MACRO: median (IQR): 200 (60 – 357) days | Time to first AECOPD | Low |
| Horsfall 2011  United Kingdom | 504206 | UK primary care database, aged 20 years or older with at least 1 bilirubin test | Male: 218727 (43.4)  Current smokers: 112559 (22.3)^1^  Age (mean): 54.6 years^1^  Race: NR  FEV1 (mean): NR  Serum Bilirubin – mean: 0.62 mg/dL^1^  UL Bilirubin: women: 1.75 mg/dL, men: 2.34 mg/dL | Median (IQR): 8 (4-11) years | COPD incidence  Mortality | Low |
| Leem 2018  South Korea | 7986 | Ansung-Ansan cohort study. Korean community based cohort study of urban and rural Koreans aged 40-69 y | Male: 3862 (48.4) ^1^  Current smokers: 1996 (25.0) ^1^  Age (mean): 51.9 years^1^  Race: NR  FEV1 (mean): 2.92 L^1^  Serum Bilirubin – mean: 0.60 mg/dL^1,4^  UL Bilirubin: women: 1.75 mg/dL, men: 2.34 mg/dL | Median: 3.6 years | FEV_1_ mL/yr  FEV_1_/FVC ratio change/yr | Low |
| Leem 2019  South Korea | 535 | Korean obstructive lung disease (KOLD) cohort, South Korea, FEV1/FVC < 0.7, age > 40, > 10 pack years, no or minimal abnormality on chest xray | Male: 519 (97.0)  Current smokers: 191 (35.8)  Age – mean (SD): 67.9 (7.9) years  Race: NR  FEV1 – mean (SD): 56.8 (16.6) % predicted  Serum Bilirubin – mean (SD): 0.68 (0.29) mg/dL  UL Bilirubin: women: 1.75 mg/dL, men: 2.34 mg/dL | Mean (SD): 5.4 (3.7) years | FEV_1_  FEV_1_/FVC ratio  6MW  CAT  SGRQ  AECOPD/year  Mortality | Mod. |
| MacDonald 2019  20 countries | 903 | Secondary analysis of START Pulmonary Substudy; untreated HIV positive participants with CD4 > 500 on entry | Male: 649 (71.9)  Current smokers: 257 (28.5)  Age – median (IQR): 36 (30, 44) years  Race: Black 345(38.2), Latino/Hispanic 158(17.5),  Asian 87(9.6), White 304(33.7), Other 9(1.0)  FEV1 – median (IQR): 96.6 (86.0, 104.0) % predicted  Serum Bilirubin – median (IQR): 0.50 (0.40, 0.70) mg/dL  UL Bilirubin: no UL | Median: 3.9 years | FEV_1_ (mL/yr)  FEV_1_/FVC decline | Low |
| **Cross-sectional studies** | | | | | | |
| Curjuric 2014  Switzerland | 4195 | Cohort study of 8 Swiss communities, aged 18-60 years | Male: 1965 (46.8)  Current smokers: 929 (22.1)  Age – mean (SD): 51.9 (11.5) years  Race: NR  FEV1 – mean (SD): 3.2 (0.8) L  Serum Bilirubin – median (IQR): 0.40 (0.29, 0.59) mg/dL^4^  UL Bilirubin: > 0.99 mg/dL set to missing | N/A | FEV_1_  FEV_1_/FVC ratio | Mod. |
| Horsfall 2014  England, Wales, and Scotland | 1551 | National Survey of Health and Development cohort: socially stratified sample of 5362 births in England, Wales, and Scotland in one week in 1946 | Male: 1092 (50)  Current smokers: 785 (35.8)  Age: 53 years^3^  Race: NR  FEV1 – mean (SD): 3.51 (0.90) L  Serum Bilirubin – mean (SD): 0.56 mg/dL^1,4^  UL Bilirubin: no UL | N/A | FEV1 | Mod. |
| Lee 2018  South Korea | 131 | Non-smoking participants in the CODA (COPD in dusty areas) Cohort; South Korean adults who live near cement plants | Male: 36 (27.5)  Current smokers: 0 (excluded)  Age – median (IQR): 73.0 (70, 77) years  Race: NR  FEV1 – median (IQR): 94.0 (80.6, 104.5) % predicted  Serum Bilirubin – median: 0.76 mg/dL^1^  UL Bilirubin: no UL | N/A | COPD | High |
| Melevoj Kopcinovic 2016  Croatia | 151 | COPD patients from single pulmonary department in Croatia  Healthy volunteers from same geographic area | Male: 100 (66.2)^1^  Current smokers: 48 (31.7)^1^  Age – median (IQR):  51 (39-84) in 45 healthy controls  71 (39-83) in 106 with COPD  Race: NR  FEV1 – mean: 59.8% predicted^1^  Serum Bilirubin – median (IQR) mg/dL^4^:  0.42 (0.32, 0.63) in 45 healthy controls  0.37 (0.30, 0.49) in 106 with COPD  UL Bilirubin: no UL | N/A | COPD | High |
| Schunemann 1997  United States | 132 | Controls from a case-control study. Non-smoking, aged 35-79, from 2 counties in New York, United States | Male: 81 (61.3)  Current smokers: 0 (excluded)  Age – mean: 58.2^1^  Race: White 132 (100)  FEV1 – mean: 105.7^1^  Serum Bilirubin – mean: 0.64 mg/dL^1^  UL Bilirubin: no UL | N/A | FEV_1_ | High |
| Wei 2015  China | 129 | COPD patients from a single pulmonary clinic  Controls from other clinics at the same hospital | Male: NR  Current smokers: 83 (64.3)  Age – mean: 62.6 years^1^  Race: NR  FEV1 – mean: 105.7 % predicted^1^  Serum Bilirubin – NR  UL Bilirubin: no UL | N/A | COPD | High |
| Yang 2015  United States | 12996 | NHANES III, > 18 years of age with WBC, FEV1, laboratory data, and clinical exams. Excluded asthma, chronic bronchitis/emphysema, and WBC > 12K or < 4K. | Male: 6220 (47.9)  Current smokers: 3217 (24.8)  Age – mean (SD): 46.16 (19.66) years  Race: Non-Hispanic White 5253(40.4)  FEV1 – mean (SD): 97.21 (16.6) % predicted  Serum Bilirubin – mean (SD): 0.59 (0.33) mg/dL  UL Bilirubin: no UL | 3217 (24.8) | FEV_1_ | High |

FEV_1_, forced expiratory volume in 1 second; UL, upper limit; FVC, forced vital capacity; AECOPD, acute exacerbations of chronic obstructive pulmonary disease; NR, not reported; IQR, interquartile range; 6MW, 6 minute walk test; CAT, COPD assessment test; SGRQ, St. George’s Respiratory Questionnaire; HIV, human immunodeficiency virus; STATCOPE, Simvastatin for the Prevention of Exacerbations in Moderate-to-Severe COPD; MACRO, Macrolide Azithromycin to Prevent Rapid Worsening of Symptoms Associated with Chronic Obstructive Pulmonary Disease; START, Strategic Timing of Antiretroviral Therapy; NHANES, National Health and Nutrition Examination Survey; WBC, white blood cell count

1: Calculated from data presented in manuscript

2: No median follow-up was reported, participants were followed for up to 9 years after initial pulmonary function testing

3: Included only active participants at age 53, data from the same participants was also analyzed from age 43 and age 63 visits, but no longitudinal analysis was performed

4: Converted from umol/L to mg/dL using factor of 0.0585

**Additional file 1: Table 2. Newcastle-Ottawa Scale Risk of Bias Assessment**

| **Cohort studies** | Selection | | | | Comparability | | Outcome | | | Total |
| --- | --- | --- | --- | --- | --- | --- | --- | --- | --- | --- |
|  | Representative-ness of exposed cohort | Selection of non-exposed cohort | Ascertainment of exposure | Outcome not present at start | Controls for smoking | Controls for additional factor | Assessment | Follow-up Duration | Adequacy of follow-up |  |
| Brown, 2017 | * | * | * | * | * | * | * | * | * | 9 |
| Leem, 2018 | * | * | * | * | * | * | * | * | * | 9 |
| MacDonald, 2018 | * | * | * | * | * | * | * | * | * | 9 |
| Horsfall, 2011 | * | * | * | * | * | * | * | * | * | 9 |
| Horsfall, 2014 | * | * | * |  | * | * | * | * |  | 7 |
| Lee, 2018 | * | * | * |  |  | * | * |  | * | 6 |
| Schünemann, 1997 | * | * | * |  | * | * | * |  |  | 6 |
| Apperley, 2015 | * | * | * | * | * | * | * | * | * | 9 |
| Curjuric, 2014 | * | * | * |  | * | * | * |  | * | 7 |
| Yang, 2015 | * | * | * | * |  |  | * |  |  | 5 |
| Leem, 2019 |  | * | * |  | * | * | * | * | * | 7 |
|  |  |  |  |  |  |  |  |  |  |  |
| **Case-control studies** | Selection | | | | Comparability | | Exposure | | | Total |
|  | Adequacy of case definition | Representative-ness of the cases | Selection of controls | Definition of controls | Controls for smoking | Controls for additional factor | Ascertainment of exposure | Same method of ascertainment for case and controls | Non-response rate |  |
| Wei, 2015 | * | * | * |  |  |  | * | * | * | 6 |
| Milevoj Kopčinović, 2016 | * |  |  |  |  |  | * | * | * | 4 |

**Additional file 1: Table 3. Summary of Findings and Strength Of Evidence**

| **Comparison** | **Outcome** | **#Observational Studies**  **(ROB:L/M/H) (n=analyzed)** | **Finding** | **Study Limitations** | **Consistency** | **Directness** | **Precision** | **Overall SOE** |
| --- | --- | --- | --- | --- | --- | --- | --- | --- |
| “Higher” vs. “Lower” serum bilirubin | Lung function (FEV_1_) | 8  (3/4/1)  (n=32,978) | Favors “higher” bilirubin | Medium | Inconsistent | Direct | Imprecise | **Low** |
|  | COPD diagnosis | 4  (1/0/3)  (n=504617) | Favors “higher” bilirubin | Medium | Inconsistent | Direct | Imprecise | **Low** |
|  | FEV1/FVC ratio | 4  (2/2/0)  (n=13619) | Unknown | Medium | Inconsistent | Direct | Imprecise | **Insufficient** |
|  | Acute COPD exacerbation | 2  (1/1/0)  (n=2,406) | Favors “higher” bilirubin | Medium | Consistent | Direct | Precise | **Low** |
|  | Mortality | 3  (2/1/0)  (n=509,421) | Favors “higher” bilirubin | Low | Inconsistent | Direct | Imprecise | **Low** |
|  | Quality of Life and Exercise Capacity | 1  (0/1/0)  (n=535) | Unknown | Moderate | Unknown | Direct | Imprecise | **Insufficient** |

ROB, risk of bias; L, low; M, moderate; H, high; FEV_1_, forced expiratory volume in 1 second; FVC, forced vital capacity; COPD, chronic obstructive pulmonary disease

**Additional file 1: References**

1. Jones PW. St. George’s Respiratory Questionnaire: MCID. *COPD J. Chronic Obstr. Pulm. Dis.* 2005; 2: 75–79.

2. Kon SSC, Canavan JL, Jones SE, Nolan CM, Clark AL, Dickson MJ, Haselden BM, Polkey MI, Man WDC. Minimum clinically important difference for the COPD Assessment Test: A prospective analysis. *Lancet Respir. Med.* Elsevier Ltd; 2014; 2: 195–203.

3. Singh SJ, Puhan MA, Andrianopoulos V, Hernandes NA, Mitchell KE, Hill CJ, Lee AL, Camillo CA, Troosters T, Spruit MA, Carlin BW, Wanger J, Pepin V, Saey D, Pitta F, Kaminsky DA, McCormack MC, MacIntyre N, Culver BH, Sciurba FC, Revill SM, Delafosse V, Holland AE. An official systematic review of the European Respiratory Society/American Thoracic Society: Measurement properties of field walking tests in chronic respiratory disease. *Eur. Respir. J.* 2014; 44: 1447–1478.

4. Albert RK, Connett JE, Bailey WC, Casaburi R, Cooper JAD, Criner GJ, Curtis JL, Dransfield MT, Han MK, Lazarus SC, Make BJ, Marchetti N, Martinez FJ, Madinger NE, McEvoy C, Niewoehner DE, Porsasz J, Price CS, Reilly J, Scanlon PD, Sciurba FC, Scharf SM, Washko GR, Woodruff PG, Anthonisen NR. Azithromycin for the Prevention of Exacerbations of COPD. *N. Engl. J. Med.* 2011; 364: 689–698.

5. Criner GJ, Connett JE, Aaron SD, Albert RK, Bailey WC, Casaburi R, Cooper JAD, Curtis JL, Dransfield MT, Han MK, Make B, Marchetti N, Martinez FJ, Niewoehner DE, Scanlon PD, Sciurba FC, Scharf SM, Sin DD, Voelker H, Washko GR, Woodruff PG, Lazarus SC. Simvastatin for the prevention of exacerbations in moderate-to-severe COPD. *N. Engl. J. Med.* 2014; 370: 2201–2210.

6. Martinez FJ, Calverley PMA, Goehring UM, Brose M, Fabbri LM, Rabe KF. Effect of roflumilast on exacerbations in patients with severe chronic obstructive pulmonary disease uncontrolled by combination therapy (REACT): A multicentre randomised controlled trial. *Lancet* Elsevier Ltd; 2015; 385: 857–866.

7. Anthonisen NR, Connett JE, Kiley JP, Altose MD, Bailey WC, Buist AS, Conway WA, Enright PL, Kanner RE, Hara PO, Owens GR, Scanlon PD, Tashkin DP, Wise RA, Conway Jr. WA, Enright PL, Kanner RE, O’Hara P, et al., Nr A, Je C, Jp K, Wc B, As B, Jr CWA, Pl E, Anthonisen NR. Effects of Smoking Intervention and the Use of an Inhaled Anticholinergic Bronchodilator on the Rate of Decline of FEV1. *Jama* 1994; 272: 1497–1505.

8. Kerstjens HAM, Rijcken B, Schouten JP, Postma DS. Decline of FEV 1 by age and smoking status: facts, figures, and fallacies. *Thorax* 1997; 52: 820–827.

9. Bhatt SP, Balte PP, Schwartz JE, Cassano PA, Couper D, Jacobs DR, Kalhan R, O’Connor GT, Yende S, Sanders JL, Umans JG, Dransfield MT, Chaves PH, White WB, Oelsner EC. Discriminative Accuracy of FEV1:FVC Thresholds for COPD-Related Hospitalization and Mortality. *JAMA - J. Am. Med. Assoc.* 2019; 321: 2438–2447.
